# Proteomic study of advanced cirrhosis based on HCV to reveal potential biomarkers 

**Published:** 2020

**Authors:** Akram Safaei, Afsaneh Arefi Oskouie, Seyed Reza Mohebbi, Zahra Razaghi, Naser Nejadi

**Affiliations:** 1 *Proteomics Research Center, Faculty of Paramedical Sciences, Shahid Beheshti University of Medical Sciences, Tehran, Iran*; 2 *Faculty of Paramedical Sciences, Shahid Beheshti University of Medical Sciences, Tehran, Iran*; 3 *Gastroenterology and Liver Diseases Research Center, Research Institute for Gastroenterology and Liver Diseases, Shahid Beheshti University of Medical Sciences, Tehran, Iran*; 4 *Laser Application in Medical Sciences Research Center, Shahid Beheshti University of Medical Sciences, Tehran, Iran*

**Keywords:** Proteomic, liver cirrhosis, hepatitis C, Two-dimensional gel electrophoresis (2DE), Protein –protein interaction

## Abstract

**Aim:**

We aimed to carry out proteomic assessment of long-term effects of hepatitis C on liver.

**Background:**

Cirrhosis is a condition where liver is damaged and loses its efficiency, and has the high rate of mortality in the world. Proteome profiling may help to identify important proteins and find the pathogenesis Cirrhosis is a condition where liver is damaged and loses its efficiency, and has the high rate of mortality in the world. Proteome profiling may help to identify important proteins and find the pathogenesis.

**Methods:**

Here, by the application of two-dimensional polyacrylamide gel electrophoresis (2-D PAGE), combined with (MALDI-TOF-TOF MS), proteome profile of decompensated HCV cirrhosis is determined compared to healthy matched controls. Furthermore, Cytoscape has used network analysis. The proteome comparison between two groups identified proteins with significant expression changes (p<0.05 and fold change ≥ 1.5).

**Results:**

We found upregulation of IGHA1, C3, A1BG, IGKC and one isoform of HP. Also, lower expression of APOA4 and the other spot of HP in advanced cirrhosis patients were revealed based on HCV compared to matched controls. According to network analysis, ALB has been introduced as a key protein, which may play an important role in pathogenesis.

**Conclusion:**

Integration of the proteomics with protein interaction data led to the identification of several novel key proteins related to the immune system that may reflect the long-term effects of hepatitis C virus on the liver, and can introduce as therapeutic targets for advanced HCV- cirrhosis.

## Introduction

 Cirrhosis is a condition where liver loses its function. This disease is followed by different chronic liver disorders such as hepatitis (1). In cirrhotic patients, the damaged liver cell is replaced by collagen layers in response to inflammatory reactions through chronic liver injury (2, 3). In addition, hepatocellular carcinoma usually accrue in 80% cases of cirrhosis (4). In advanced cirrhotic patients, the mortality without liver transplantation is as high as 85% over 5 years (3). A method to inhibit the cirrhosis development based on hepatitis is interferon-based therapy that has a number of challenges including toxicity and poor tolerability. Also, Direct-Acting Antiviral (DAA) therapy is not an exhaustive treatment because of the deficiency response of some subgroup of HCV to DAA therapy (5).  In fact, appropriate treatment for decompensated cirrhosis is liver transplantation (6) and many advanced cirrhotic patients may die while awaiting liver transplantation because of the shortage of organ donors (7). Complexity in the treatment of HCV advanced cirrhosis remains a significant challenge so,  it is important to know the molecular aspects and etiology of cirrhosis, since it can enhance understanding of the pathogenesis and provide valuable information for direct treatment decisions (8). Proteomics is the term used for complete analysis of protein structure and function in an organ or tissue (9). The ability of proteomics to compare differences in proteome profiles has been adapted in clinical research for the biomarker identification (10-15), and it is also useful for the elucidation of the protein alterations and discovery of liver diseases markers (16). Serum can be used as a less invasive method that is easy collection. In fact, the identified biomarker in serum which is associated with the pathology disease can be easily applied for the follow-up treatment or diagnosis of diseases (17). In this study, the serum-based proteomic approach has been applied to identify new biomarkers and then it performs network PPI analysis of significantly changed proteins. We hoped to learn more about the mechanisms involved in the pathogenesis of advanced cirrhosis, and also find the treatment biomarker(s) that might improve diagnosis and therapy of advanced hepatic cirrhosis based on HCV. 

## Methods

**Patients and collection of serum samples**

The sample collection of decompensated HCV-cirrhosis was carried on in Gastroenterology and Liver Diseases Research Center of Taleghani Hospital and Loghman Hakim Hospital, Shahid Beheshti University of Medical Sciences. In total, 21 decompensated HCV-cirrhosis and 18 healthy blood samples were obtained preoperatively from November 2014 to July 2015. Control blood samples were obtained from 18 healthy volunteers without history and current diseases, a habit of alcohol consumption and smoking. Decompensated cirrhosis was defined as the presence of the following five criteria: hypoalbuminemia, hyperbilirubinemia, ascites, peripheral edema of noncardiac or renal origin (18) and were evaluated according to clinical examinations. The baseline clinical characteristics of the cirrhotic patients and controls are summarized in Table 1. All patients were positive for hepatitis C antigen for anti HCV antibody and HCV RNA RT- PCR. The severity of liver disease was calculated according to the model for end-stage liver disease (19). We excluded patients with the past or current hepatocellular carcinoma, NAFLD, NASH, alcoholic, diabetes, cardiovascular disease, and kidney disease and any other viral infection, hepatitis delta or hepatitis B virus. 

**Table 1:** Characteristics of patients with Decompensated HCV- cirrhosis^ a^

Parameters	Control	Decompensated HCV- cirrhosis
Age (year)	50±2.0	56±1.2
Male Female	14 4	17 4
ALT (U/L)	29.5 ±4	81.4±2.6
AST (U/L)	23.1 ±11.6	137.8 ±53.9
Total bilirubin (µmol/L)	16.9 ±3.6	46.1 ±10.7
ALB (g/L)	45.8 ±10.2	30 ±11.2
INR	1.0 ±0.15	1.4 ±0.1
TP (g/L)	69 ±9.6	58 ±5.7
MELD score	------	14.5±6.5

^a ^Data are expressed as mean values ± standard deviation. AST = aspartate aminotransferase; ALT = alanine aminotransferase; ALB=albumin; INR = international normalized ratio; TP= total protein, MELD= model for end-stage liver disease

Blood collection was handled using a needle with gauge 2°C.  After clotting in the room temperature for 30 min, serum samples were completely separated by centrifuging two times at 4ºC with 2000 g and 10 min duration. Then, the micro tubes containing serum samples were kept at −80°C until use.

**Two-dimensional electrophoresis (2-DE)**

2D-electrophoresis materials were provided by SERVA Company (http://www.serva.de) and GE HealthCare Life Sciences (http://www.gelifesciences.com). We have two groups for analysis, proteins from decompensated HCV-cirrhosis samples and healthy matched controls that was pooled each group, separately. Proteins were extracted using the 2-DE Clean-Up Kit (GE Healthcare). Protein concentrations were determined using the 2-DE Quant Kit (GE Healthcare). Prior to IEF, IPG strips were passively rehydrated in the presence of 1mg for 8 hours. Bio-Rad PROTEAN IEF Cell, 11-cm nonlinear IPG with pH range 4-7 was used for the first dimension (Isoelectric Focusing (IEF)) to separate proteins based on their PH Isoelectric; it took 7.5 hours at 20 °C. Following that, IPG strips were then equilibrated for 30 min at room temperature in the equilibration solution (Serva Kit). Second dimension was SDS-PAGE. In this stage, the protein separation was based on MW by the application of the HPE FlatTop Tower (horizontal electrophoresis) using 2D HPE™ Double-Gel 12.5 % Kit (Serva Company) for about 210 minutes. This step is followed by staining through the application of SERVA HPE™Coomassie® Staining Kit according to the protocol. 

**Image analysis and spot selection**

Spots were scanned using a calibrated GS-800 densitometer (Bio-Rad) scanner. Prognosis Same-Spots Software, as an image analyzer, detected protein expression changes by comparing healthy control and decompensated HCV-cirrhosis gels. Spots that had 1.5 or more than 1.5-fold changes with p<0.05 were considered as a differential expression variation and selected for the analysis by mass spectrometry. 

**Protein identification of advanced cirrhosis based HCV**

Statistically significant differences (*p *≤ 0.05) in spot intensity were identified by one-way ANOVA analysis.  After selecting significantly changed proteins (Fold change ≥1.5 and P-value<0.05), MALDI-TOF /MS was used to evaluate the candidate spots. In way that, prior to treatment with trypsin, they were destained and subjected to dithiotreitol (DTT) and iodoacetamide for reduction and alkylation, respectively. Then, the extracted peptides were assessed by MS and the spectra were submitted to MASCOT (http://www.matrixscience.com) for protein identification.

**Network topological properties**

In network analysis, there are several important topological indicators that have been defined to describe the characteristics of the nodes in a network. The most representative ones are the degree and betweenness centrality (BC). The degree is defined as the number of nodes interacting with other nodes. BC of a node is defined as the number of shortest paths (the nearest distance traveling from one node to another) going through the node and the nodes with high BC introduced as bottleneck (20). In this study, the proteins with the high degree were considered as key proteins named hub. We selected 20% of nodes in PPI network with a high degree. The hubs with BC>0.05 has neen regarded as hub-bottelneck. CluGO is a plug-in apps in Cytoscape used for the analysis of biological terms of proteins. It can analyze genes according to functionally grouped terms and comprehensively visualize them. The significant functional enrichment was quantitatively assessed (21). The applied criteria for annotation analysis of differentially changed proteins included Kappa statistic ≥ 0.5 and Bonferroni step-down method for probability value correction. 

## Results

**Differences in the proteome of advanced cirrhosis based HCV compared to healthy subjects**

**Figure 1:**
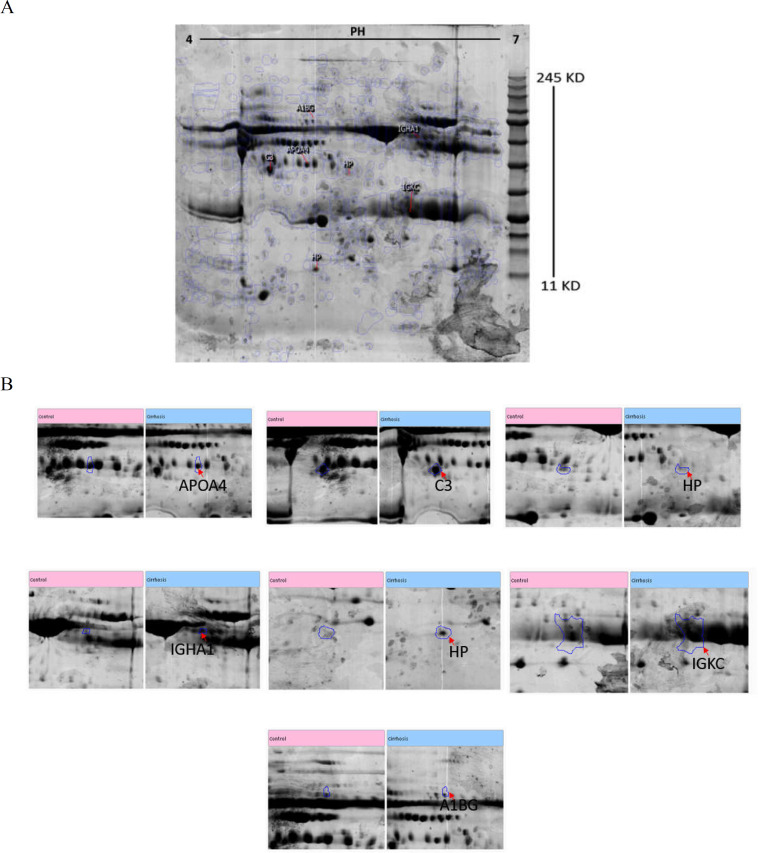
Serum proteins separated by two-dimensional gel electrophoresis in advanced cirrhosis based on HCV. A) Serum profiles separated by 2D gel electrophoresis from each group (control and patient), that were analyzed using the same spot software. 7Seven spots were characterised by MS after excision. Spots include APOA4, IGHA1, A1BG, IGKC, C3 and 2 spots are related to HP. (this gel is related to cirrhosis group considered as referenced gel in this study). B) The reports of same spots software for seven differentially expressed proteins of advanced HCV-cirrhosis versus normal matched control

 Identification of differentially abundant proteins were separated by the 2D gel and analyzed by MS after in-gel digestion. In total, ninety-six protein spots were identified twenty-two different proteins reveal that were differentially expressed between the patients and the controls (P < 0.05; fold change ≥ 1.5). The selected protein spots were excised and subjected to in-gel tryptic digestion. The extracted peptides were analyzed by MALDI TOF. Seven selected spots are summarized in Table 2. Figure 1 shows differently expressed spots in patients and healthy controls. Two spots in proteins showed an over-expression in cirrhotic sera, whereas 5 proteins showed a lower expression. The expression of Ig alpha -1 chain C region, Ig kappa chain C region, alpha 1-B glycoprotein and complement 3 and one of the spots of haptoglobin was significantly higher in advanced HCV-cirrhotic septa. Additionally, 1 spot of haptoglobin and apo lipoprotein 4 show lower lever of expression in advanced HCV- cirrhosis (Supporting Table 2).

**Table 2:** The key proteins in the protein–protein interaction network of decompensated HCV-cirrhosis. The data are sorted according to degree score from largest to smallest. The asterisked nodes are introduced as hub-bottleneck (high degree and BC).The cut-off for BC is ≥ 0.05

BC	Degree	Gene Name	Protein Name	
0.08367	38	ALB*	Albumin	
0.07169	37	HP*	Haptoglobin	
0.03665	30	TSPO	Translocator protein (18kDa)	
0.03467	29	PRDM10	PR domain containing 10	
0.01734	27	APOA1	Apolipoprotein A-I	
0.02253	26	APOE	Apolipoprotein E	
0.02030		26	TF	Transferrin
0.01539		26	IL6	Interleukin 6 (interferon, beta 2)

**PPI network and key proteins identification of advanced cirrhosis based HCV**

**Figure 2:**
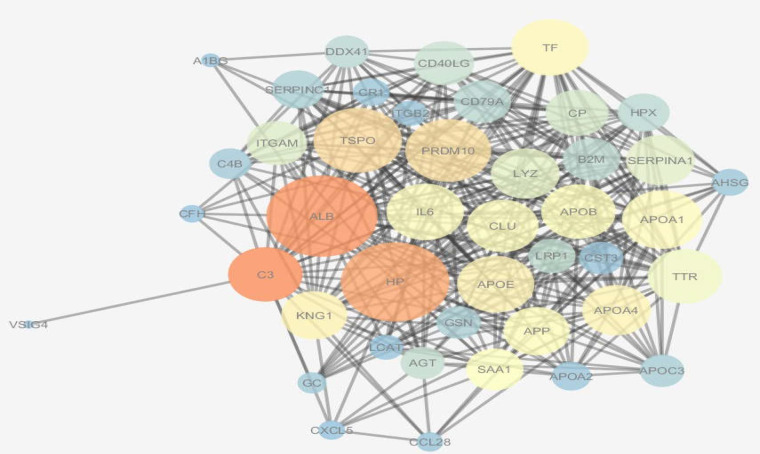
Protein-protein interaction network of differentially expressed proteins of decompensated HCV- cirrhosis contain 43 nods and 394 edges. The larger circles correspond to the higher degrees and blue to brown color refers to increment of betweenness value which in this network is the darkest color and largest size that reflects the most important protein which belong to ALB

Cytoscape software version 3.4 was used for PPI network construction (22). Topological centralities (degree and BC) were evaluated to distinguish the biological value of proteins. The final network was visualized based on degree and BC wherein we mapped the node degree to the node size and BC value according to color (figure 2). The PPI network obtained contain 43 nods and 394 edges. The hub nodes included ALB, HP, TSPO, PRDM10, APOE, APOA1, TF and IL6 (table 2). In figure 2, the larger circles correspond to the higher degrees, and blue to brown color refers to the increment of BC. According to table 3, the functional enrichment for differentially expressed proteins is complement, classical activation pathway, and functional enrichment for whole PPI of differentially expressed proteins is reverse cholesterol transport. Blood micro particle is the most significant cellular component in both whole PPI of differentially expressed proteins and differentially expressed proteins. Cholesterol binding is a high-score term despite no GO term for molecular function of differentially expressed proteins. 

**Table 3:** GO functional enrichment analysis for differentially expressed proteins and PPI of differentially expressed proteins of decompensated HCV- cirrhosis

Differentially expressed proteins	GO term	Description	Genes	P-value
	BP	Complement, classical activation pathway	C3,IGKC,IGHA1	2.4E-6
	CC	Blood micro particle	C3,IGKC,IGHA1,HP,A1BG,APOA4	3.0E-13
	MF	No GO term	---------	-------
PPI of differentially expressed proteins	GO term	Description	Genes	P-value
	BP	Reverse cholesterol transport	APOA1,APOA2,APOA4,APOC3,APOE,CLU,LCAT	3.0E-13
	CC	Blood micro particle	A1BG, AGT, AHSG, APOA1, APOA2, APOA4, APOE, C3, CLU,CP,GC,HPX,KNG1,TF	2.1E20
	MF	Cholesterol binding	APOA1,APOA2,APOA4, APOC3, APOE,TSPO	2.5E-9

**Table 3:** GO functional enrichment analysis for differentially expressed proteins and PPI of differentially expressed proteins of decompensated HCV- cirrhosis

Differentially expressed proteins	GO term	Description	Genes	P-value
	BP	Complement, classical activation pathway	C3,IGKC,IGHA1	2.4E-6
	CC	Blood micro particle	C3,IGKC,IGHA1,HP,A1BG,APOA4	3.0E-13
	MF	No GO term	---------	-------
PPI of differentially expressed proteins	GO term	Description	Genes	P-value
	BP	Reverse cholesterol transport	APOA1,APOA2,APOA4,APOC3,APOE,CLU,LCAT	3.0E-13
	CC	Blood micro particle	A1BG, AGT, AHSG, APOA1, APOA2, APOA4, APOE, C3, CLU,CP,GC,HPX,KNG1,TF	2.1E20
	MF	Cholesterol binding	APOA1,APOA2,APOA4, APOC3, APOE,TSPO	2.5E-9

## Discussion

**Potential biomarkers in decompensated HCV- cirrhosis according to proteomics results**

Hepatitis leads to the destruction of liver tissues and consequent complications that lead to hepatic cirrhosis, which is the major risk factor for the development of HCC (23). Currently, lack of robust biomarkers still limits the organization of hepatic fibrosis stages, especially disease associated with HCV infection (24). Liver biopsy remains the gold standard for the assessment of hepatic fibrosis, so biomarkers that facilitate early detection and early treatment of advanced HCV cirrhosis are essentially needed (25). For this purpose, we analyzed human cirrhotic sera by a proteomic approach and elevated network analysis of significantly changed proteins in our patients versus controls. Haptoglobin is one of the deregulated serum proteins detected in advanced HCV- cirrhosis (table1). An HP spot show increased expression and the other spot of HP show a lower expression (figure 1A and B). This observation is in accordance with previous studies that have demonstrated an association of hepatitis with the appearance of haptoglobin isoforms (26). Haptoglobin is a key player in the maintenance of free haem in the blood and thus protects cells from its oxidative effects. Plasma haptoglobin depletion is considered as a marker for the diagnosis of the presence of inflammation or accelerated red cell destruction (haemolysis) (27). The low concentrations of serum haptoglobin found in liver cirrhosis are probably due to the increased breakdown of erythrocytes in the enlarged spleen of patients with portal hypertension(28). The decrease of haptoglobin in serum cirrhosis patients has also been reported (29). Meanwhile, Mondal G reported elevated level of haptoglobin in HCV-cirrhosis and HCC patients' group with respect to control(30). Another study showed that beta chain of haptoglobin decrease in the plasma of cirrhosis based on HCV (31). Total haptoglobin along with other proteins is an acceptable marker to diagnose liver fibrosis and also known to decrease in fibrosis (32) . Our data beside the mentioned previous studies shows that although haptoglobin may be introduced as a fibrosis marker, the isoforms may show a different panel for deregulation in advanced HCV-cirrhosis (figure 1). Apolipoprotein A4 is the glycoprotein in our study which shows down-regulated expression in advanced cirrhosis based on HCV. It has not only been identified as suggested serum discriminator of fibrosis but also as a marker in different types of cancer (33). It has been reported that the depletion in level of plasma lipoproteins related with the severity of liver disease and activity of enzymes is involved in lipid metabolism (34). In addition, HCV infection induces expression of lipid metabolism genes (35). Our hypothesis is that the decreased apolipoprotein A4 levels in advanced cirrhosis-based HCV might be explained by changes in the synthesis of enzyme in lipid metabolism. Complement C3 is another protein that is upregulated in our patients. Impairment in complement function will contribute to the impaired antibacterial host defense of the patient with chronic hepatic disease based on hepatitis (36). Additionally, C3 deficiency in patients with alcoholic cirrhosis predisposes the infection and so increased mortality(37). Baumann and colleagues showed that serum complement concentrations of C3 and C4 correlated negatively with the severity of liver cirrhosis. In contrast to our findings, they showed that C3 concentration is lower in Child-Pugh C cirrhosis patients (decompensated cirrhosis)(38). The other differentially expressed protein is IGKC that encodes the constant domain of kappa-type light chains for antibodies  and was a member of the most overexpressed genes involved in antigen presentation in cirrhosis based on alcoholic and HCV (39). Our observation of the serum of HCV-cirrhosis is in accordance with previous studies that have demonstrated an association of HCV- cirrhosis with the upregulation of Ig kappa chain C region (40). 

**Potential biomarkers in decompensated HCV- cirrhosis according to network analysis**

Potential interactions between all the differentially expressed proteins were analyzed using Cytoscape software. A network diagram showing potential interactions between the identified changed proteins is shown in Figure 2. Interestingly, the software demonstrated that some of the identified proteins were related to albumin which is the main factor in the management of complications of liver cirrhosis (41). It has been reported that the hubs are affected in diseases based on HCV infection (30, 39, 42-44). ALB has the largest degree and BC in PPI network of advanced cirrhosis based on HCV. APOA1 and APOE are the other key proteins in network analysis. According to our previous network analysis of differentially expressed proteins in cirrhosis serum based on literatures, APOA1 and APOE have been introduced as key proteins (hub) in cirrhosis (45). APOA family is involved in the most significant biological process (table 4) and thus may implicate the importance of their roles in decompensated cirrhosis based on HCV development similar to cirrhosis based on fatty liver disease (46). Top associated functions of differentially expressed proteins were complement, classical activation pathway (P=2.4 E-6) (table 3). GO analysis showed that the identified proteins were most closely related to immune response pathways involved in the complement activation which is consistent with the hepatic scarring process since inflammation has an association with virus infection. In fact, these results suggest an inflammatory response that provides protection against the viral infection using immune defense mechanisms (47). According to previous results, the finding pathways for differentially expressed proteins (complement, classical activation pathway ) and PPI for differentially expressed proteins (reverse cholesterol transport ) can be associated with cirrhosis disease (48, 49) that may provide a means for the development of molecularly targeted therapies for HCV-induced cirrhosis. 

In summary, these results identify significant differences in proteins in advanced HCV cirrhosis serum compared to control, and that these differences can be identified by a proteomic approach. Additionally, the protein species identified in this study provide further insight into possible pathophysiologic factors in HCV cirrhosis, including changes in the expression of proteins in patients involved in antigen presentation and complement, classical activation pathway. Further investigation is warranted in a larger number of patients in order to confirm the differentially proteins identified in this study.
